# Cancer risk in persons with HIV/AIDS in India: a review and future directions for research

**DOI:** 10.1186/1750-9378-4-4

**Published:** 2009-03-28

**Authors:** Robert J Biggar, Anil K Chaturvedi, Kishor Bhatia, Sam M Mbulaiteye

**Affiliations:** 1 Dept. of Research, LV Prasad Eye Hospital, Hyderabad, India; 2 Department of Epidemiology Research, State Serum Institute, Copenhagen, Denmark; 3 Infection and Immunoepidemiology Branch, Division of Epidemiology and Genetics, National Cancer Institute, Bethesda, Maryland, USA; 4 AIDS Malignancy Program, National Cancer Institute, Bethesda, Maryland, USA

## Abstract

**Background:**

India has a large and evolving HIV epidemic. Little is known about cancer risk in Indian persons with HIV/AIDS (PHA) but risk is thought to be low.

**Methods:**

To describe the state of knowledge about cancer patterns in Indian PHA, we reviewed reports from the international and Indian literature.

**Results:**

As elsewhere, non-Hodgkin lymphomas dominate the profile of recognized cancers, with immunoblastic/large cell diffuse lymphoma being the most common type. Hodgkin lymphoma is proportionally increased, perhaps because survival with AIDS is truncated by fatal infections. In contrast, Kaposi sarcoma is rare, in association with an apparently low prevalence of Kaposi sarcoma-associated herpesvirus. If confirmed, the reasons for the low prevalence need to be understood. Cervical, anal, vulva/vaginal and penile cancers all appear to be increased in PHA, based on limited data. The association may be confounded by sexual behaviors that transmit both HIV and human papillomavirus. Head and neck tumor incidence may also be increased, an important concern since these tumors are among the most common in India. Based on limited evidence, the increase is at buccal/palatal sites, which are associated with tobacco and betel nut chewing rather than human papillomavirus.

**Conclusion:**

With improving care of HIV and better management of infections, especially tuberculosis, the longer survival of PHA in India will likely increase the importance of cancer as a clinical problem in India. With the population's geographic and social diversity, India presents unique research opportunities that can be embedded in programs targeting HIV/AIDS and other public health priorities.

## Background

**Figure 1:**
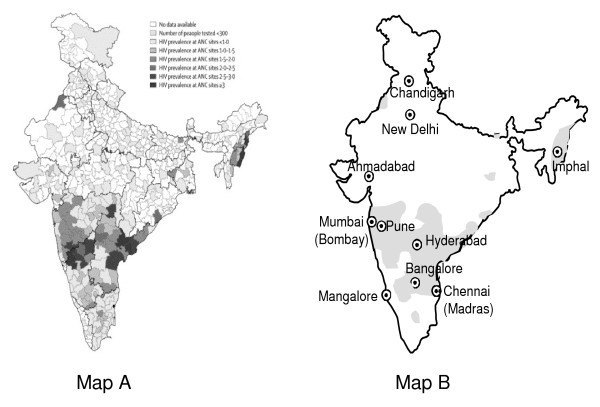
**High HIV prevalence areas of India**. Map A. HIV prevalence in India, by district. (Reproduced from Chandrasekaran, et al^2^, with Journal permission.) ANC = Antenatal Clinics. Map B. Cities mentioned in the text. Grey areas indicate where the prevalence in women attending antenatal clinics was >1% in 2005 (data approximated from Map A).

In a country as large and diverse as India, the size of the HIV/AIDS epidemic is difficult to estimate. The most recent UNAIDS Report on the Global AIDS Epidemic estimated 2.5 million persons were prevalently HIV-infected as of 2007 [1]. Although HIV prevalence appears to be stable, much remains uncertain about the direction of the epidemic. Geographically, higher prevalence (2 – 3% of antenatal women) occurs in south-central and north-eastern India (Figure 1), with higher risks in migratory and underprivileged populations [2]. Transmission in adults is predominantly heterosexual but injection drug users and men who have sex with men are also major transmission groups in some areas. In this report, we summarize what is known about the cancer experience among Indian persons with HIV/AIDS (PHA).

In the developed countries, the increased cancer risk among immunosuppressed PHA is well described [3-5] (Table 1). Kaposi sarcoma (KS) and non-Hodgkin lymphoma (NHL) occur at exceptionally high incidence, with relative risks being 100s-fold above those in uninfected populations. Cervical cancer is an AIDS-defining cancer when it occurs in an HIV-infected woman, and the relative risk is increased 5- to 10-fold [3-5]. Hodgkin lymphoma is not yet accepted as an AIDS-defining cancer in HIV+ persons, although its risk is increased 10- to 15-fold in most studies [3-5]. In immunosuppressed PHA, risk of AIDS-related cancers generally increase with degree of immunosuppression [6]. However, Hodgkin's lymphoma incidence has an inverse relationship with CD4 count, in which moderate decreases in CD4 count greatly increase risk but risk decreases with severe immunosuppression [7]. Some tumors are observed more frequently in PHA because of lifestyles that expose PHA to specific carcinogens, such as lung cancer related to the high frequency of smoking in PHA [8]. A few otherwise rare tumors, such as squamous cell carcinoma of the conjunctiva [9,10] and Merkel cell carcinoma of the skin [11], also appear to be increased. Other tumors have been reported to have marginal or inconsistent increases in PHA, and their associations with level of HIV/AIDS immunosuppression are still controversial. Whether similar associations with cancer are observed in PHA in India, where infections, genetics, environmental exposures, and cancer patterns all differ from developed countries, is unknown. Few studies have been done, and most have limited data or methodological problems.

**Table 1:** Summary of cancers with increased risks in males (M) and females (F) with HIV/AIDS (PHA) in India

**Cancer sites with increased risk**	**Relative risks in developed countries**	**Correlated with CD4 loss in PHA**	Tumor-associated co-viral infections*	**Incidence in India, relative to developed countries**	**Proportional risk increase in Indian PHA **[13]		Case observations in Indian PHA**
							

					**M**	**F**	

**AIDS-defining cancers**							

Kaposi sarcoma	> 1000	++++	KSHV	Rare	No cases seen		Occasional

Non-Hodgkin lymphoma	20–350	+++	EBV	Low	17	10	Multiple

Cervical cancer	2–20	-	HPV	Very high		4	Not reported

**Non-AIDS defining cancers**							

Anus	20–50	-	HPV	Low	10	6^NS^	Not reported

Hodgkin lymphoma	3–18	Inverse	EBV	Low	4	2^NS^	Occasional

Vulva and vagina	4–8	-	HPV	High		8	Not reported

Head and neck cancers	2–3	-	HPV	Very high	1^NS^	2	Not reported

Testis	1–2	-	None	Low	2		Not reported

^NS ^Not statistically significant

* KSHV: Kaposi sarcoma herpesvirus (Human Herpesvirus 8); EBV: Epstein Barr virus; HPV: Human papillomavirus

** Other than description in risk ratio study [13].

### Current state of knowledge on HIV/AIDS and cancer in India

In a review of cancer studies in PHA in 2005 based primarily on retrospective and autopsy data, Chitale[12] pointed out the paucity of data from India. He estimated that 3 to 4% of PHA in India will have a cancer during their course, compared to 34% in the developed countries. However, cancer incidence in PHA in developed countries has been driven largely by KS in homosexual men, a group commonly co-infected with the Kaposi sarcoma-associated herpesvirus (KSHV). KS appears to be rare in Indian PHA, and thus the cancer burden in Indian PHA should be markedly lower.

A report by Dhir et al. [13] provides the only risk estimates for cancers in HIV-infected persons in India (Table 1). These authors examined HIV prevalence in persons with all types of cancer who presented at the Tata Memorial Hospital, the largest tertiary cancer referral medical center in India, during 2001–2005. They used proportional incidence ratios (PIR) to assess the likelihood that specific cancer types were associated with HIV infection, comparing the cancer distribution in PHA to that in the age- and sex-specific distribution of all cancer patients seen at the same medical center in 2002. HIV infection was found in 1.2% of cancer admissions, including 166 men and 85 women. KS was not observed, consistent with the general impression that it is rare in India. In HIV-positive men, increased PIRs were observed for NHL and Hodgkin lymphoma (HL), 17-fold (n = 63) and 4-fold (n = 9), respectively. Five cases of testicular cancer (2-fold PIR) and 10 cases of anal canal cancer were diagnosed (10-fold). In women, NHL was increased 10-fold (n = 14), while one case of HL was observed (non-significant 2-fold increase). Cervical (n = 33) and vulva/vaginal cancer (n = 2) risks were both increased (4-fold and 8-fold, respectively), and one anal canal cancer was observed (6-fold increase, not statistically significant). They also reported marginal increases in head and neck cancer in both men (46 cases, 1.3-fold, confidence interval: 0.95–1.70) and women (13 cases, 2.3-fold, 1.26–4.12). Of additional interest, they observed one conjunctival cancer, but the PIR was not reported. This report provided no data about possible risk factors such as sexual practices, smoking or chewing tobacco, or infections.

NHL appears to be the most common type of cancer reported in Indian PHA. In the Mumbai study [13], NHL constituted 38% of the cancers seen in male PHA whereas 2% of all cancers were expected on the basis of all patients in 2002. In female PHA, the proportion of NHL was lower (16%) than in males because cervical cancer predominated. Although the most common cancer, it is not a common cause of lymphadenopathy in PHA. In India, tuberculosis and other opportunistic infections are major threats to PHA and far more likely to cause lymphadenopathy than cancer. For example, Sharma et al. [14] reported on 135 consecutive PHA, mostly untreated for HIV infection, in New Delhi between 2000 and 2003. Among specifically identified illnesses, tuberculosis dominated (71%) and co-morbid fungal diagnoses, especially Candida (39%), were common. In this series, only 2 patients (1.5%) had lymphoma. In a study of lymphadenopathy using fine needle aspiration in Mangalore, Shenoy et al. [15] found lymphoma in 10% of 48 PHA with lymphadenopathy, whereas 48% were diagnosed as tuberculosis and 36% had non-specific causes.

The types of lymphoma occurring among PHA seen at Tata Memorial Hospital, Mumbai, have been examined by Agarwal et al. [16] using molecular markers to classify tumors. Among 35 PHA with lymphoma, they found 24 NHL, 7 HL and 4 plasmocytomas. The NHLs were high-grade diffuse types consistent with immunoblastic/diffuse large B cell lymphoma (14 cases) or Burkitt lymphoma (3 cases), the remainder being unspecified. The proportion of HLs and plasmocytomas in this small study was relatively high compared to that in developed countries. All 7 HL cases were EBV+, which is unusually frequent. In a study of bone marrow samples from 140 PHA by Khandekar et al. [17] in Pune, plasmacytosis was a common feature (86%). However, only one cancer, an immunoblastic lymphoma, was observed.

NHL in the central nervous system (CNS) is unusual but in the developed countries, relative risks are high in PHA, and the risks increased rapidly as CD4 counts decline [6]. Powari et al. [18] reviewed 40 CNS NHLs in Chandigarh (North India) from 1985 to 1999, but none of the 14 patients tested was HIV-infected. In a separate study at the same institute (probably including the same patients), Tiwari et al. [19] noted CNS NHL incidence increased 10-fold from the 1970s to 2000. However, HIV prevalence was low in Chandigarh throughout those years, so the increase incidence is more likely due to improving diagnostic capabilities than increased HIV/AIDS presence. In Delhi and in Bangalore, Sarkar et al. [20] summarized CNS NHL cases during 1980–2002, finding few HIV-infected patients, but neither city had a high HIV/AIDS prevalence in most of those years.

KS can occur in Indian PHA. In 1993, two reports described the same case of a 35 year old woman from Mumbai with multiple lesions who was co-infected with HIV-1 and -2 [21,22] and other isolated case reports of KS followed [23,24]. In the National AIDS Control Program, reports of KS in PHA are uncommon [25]. Gatphoh et al. [26] followed 26 PHA in Imphal over a three year period, of whom 4 developed cancer, including two KS, one NHL and one promyelocytic leukemia. In studies of specific sites, KS is occasionally reported, but it is more conspicuous by its absence. In a review of pathology submitted on skin lesion from 134 PHA in Mumbai, Lanjewar et al. [27] reported 12 (6%) cancers, including 1 KS, 9 squamous cell carcinomas, and 2 basal cell carcinomas. The majority (53%) of the patients had infectious conditions. Expected rates for these cancers were not provided. In an autopsy study of lung tissues from 97 men and 38 women dying with HIV/AIDS in Mumbai, Lanjewar and Duggal [28] found one KS, as well as one squamous cell lung cancer. None had NHL. In their review of oral lesions in PHA, Ranganathan and Hemalatha [29] reported no cases with KS in HIV/AIDS patients from Chennai. Biswas et al. [30] followed 100 PHA attending an eye clinic in Chennai between 1993 and 1998 but observed no cancers. Similarly, Gharai et al. [31] reported no eye cancers in 100 consecutive PHA attending an eye clinic in New Delhi.

In the Mumbai study [13], cervical cancer was the most common cancer (39%) in women. However, other than in this report, cervical cancer risk in PHA has not been reported in India. In Pune, Joshi et al. [32] reported on 287 consecutive HIV-infected Indian women who underwent screening, finding 6.3% to have dysplasia but none had cancer. No data were provided about expected rates of dysplasia or cervical cancer in HIV-uninfected women of a comparable age.

## Discussion

As elsewhere, cancer is a health concern in the general population of India, where approximately 750,000 cancers are diagnosed annually [33]. The current review of the data available about cancer in PHA in India highlights the paucity of information in these high-risk persons. The studies are limited in number and duration and have few outcomes. The published studies include almost no assessment of risk factors which might affect cancer risks in PHA. In the general population of India, the patterns of diagnosed cancer differ from those in the developed countries, indicating risk factors for cancer in India differ from those in developed countries. Thus, the cancer profile in Indian PHA may also differ from that in the developed countries.

While the studies we reviewed suggest that cancer is not currently a common clinical problem, it is reasonable to speculate that competing mortality from infectious problems, particularly tuberculosis and fungal infections, has truncated survival, preventing progression to severe immunosuppression when risks for common cancers like NHL and KS increase. However, India is emerging as a leader in developing affordable regimens of highly active antiretroviral therapy to control HIV replication and drugs to treat infectious conditions. The application of these treatments will reduce the effects of competing mortality and likely amplify the importance of cancer in PHA. An AIDS-cancer record linkage between available registries in overlapping areas will be of great help in monitoring the changing relationship between AIDS and cancer incidence during a time when treatments become increasingly used.

As in the developed countries, the most important AIDS cancer type in India is NHL, perhaps affecting 2% of Indian PHA [12]. Prior to effective antiretroviral therapy, about 6% of AIDS cases in developed countries were expected to have NHL, either at AIDS onset (3%) or during their AIDS course (3%) [3]. NHL risk is higher when immunosuppression is severe [6], and competing mortality from other infections in PHA will strongly affect survival time and therefore the likelihood of developing NHL. Thus, with strong competing risks of mortality, perhaps 2% of Indian PHA ever having NHL is a reasonable estimate. The lack of CNS NHL associations so far reported may be only because studies examining this question have been conducted in areas where HIV prevalence was low in those years. Additionally, CNS NHL risks increase in severely immunosuppressed persons [6], but these PHA may have limited survival because of infections. However, if risk is not increased, it would be useful to understand why. The NHL histology appears to be typical of that seen in the developed countries, with most being immunoblastic/large cell diffuse lymphomas. Based on limited data, Burkitt lymphoma is not common, which is surprising since Burkitt lymphoma occurs in PHA with relatively high CD4 count [6]. In contrast, plasmacytomas may be somewhat more frequent than expected. Relative to NHL, HL may be more frequent than expected and also more likely to be associated with EBV. However, in developed countries, HL tends to be more frequent when PHA are not profoundly immunosuppressed [7], which may explain the increased proportional frequency in a population with limited survival after AIDS onset.

In India, KS is uncommon. KSHV infection is required for KS to develop, and population variations in KS incidence seem to reflect differences in the prevalence of KSHV. Although KS can occur without severe immunosuppression, the risk increases progressively as CD4 counts decline [6]. However, given the presence of KSHV infection, KS risk is high in PHA, even when CD4 counts are only modestly low. In developed countries, KSHV occurs predominately in men who have sex with men (30% infected compared to 1–3% infected in other groups) [3]. For unexplained reasons, there appears to be little KSHV infections in India (7% in a general population [34]) or elsewhere in Asia, at least relative to Africa. Additional studies are needed to confirm this finding, especially in men who have sex with men.

Assuming it is confirmed that KSHV is uncommon in India, why would this be so? The west coast areas of India, in particular, have had many centuries of immigration and trade interaction with east and central Africa, where half or more of adults are KSHV-infected and KS is also endemic [35]. In Africa, KSHV prevalence increases rapidly in both sexes from early childhood, particularly in poorer households without access to clean water. Transmission is probably via saliva exchange [36]. Yet, in India or other areas of Asia, KSHV has not spread, despite a large population living in poor socioeconomic conditions and lack of access to water. Additionally, studies elsewhere have found pockets of KSHV in unexpected places, such as among Amerindians in the Amazon Basin [37]. Might such pockets exist elsewhere, such as among minorities in the more remote rural areas of India? Analysis of KS data from the National AIDS Control Organization may suggest places or subpopulations where KSHV is present.

Human papillomavirus is associated with a variety of anogenital cancers and appears to be an important contributor to cancer risk in India. Cervical cancer incidence is among the highest in the world, and vulva/vaginal, anal and penile cancers all have a relatively high incidence [33]. Their risk appears to be even higher in India PHAs [13]. Similar associations have been made in the developed countries, although the proportional importance is much lower [38-40]. However, the role of HIV infection and immunosuppression in cancer causation is controversial because both HPV and HIV are similarly sexually transmitted, making if difficult to exclude confounding due to sexual behaviors. In Indian PHA, studies are needed to examine sexual behaviors in the context of the occurrence of these cancers. A specific role for HIV and immunosuppression in cervical cancer occurrence is favored by reported associations between HIV infection and high grade dysplasia at these sites in the developed countries [38-40]. However, against a specific association with HIV, cervical cancer incidence was not increased in unscreened African women despite a major HIV/AIDS epidemic in this population [41]. Furthermore, the risk of invasive cervical cancer in women with AIDS does not correlate with the degree of immunosuppression [6]. One explanation is that women in areas with limited medical care have a high competing mortality and die before dysplasia can progress to cervical cancer. As Indian PHA survive longer, these cancers could become increasingly important, making screening PHA for pre-cancerous lesions a high public health priority.

Among other cancers, the 2-fold increased risk of head and neck cancer in India [13] is concerning. This cancer is one of the most common in India [33], and small relative risk increases in PHA would have important public health consequences. However, evidence of an association is weak. Notably, the increases were mainly at buccal/palatal sites which are specifically associated with chewing tobacco and betel nut rather than at pharyngeal and tonsillar sites, which are HPV-associated. In India, tobacco and betel chewing are widely practiced, albeit less frequently in recent years [42]. Specific risk factor studies will be required to clarify if the risk in Indian PHA is increased after adjustment for this exposure, which could well differ between PHA and the general population.

Cancer risk at other or unexpected sites appears to be little increased in Indian PHA. When increases are reported, much of the association is likely to be because of confounding rather than a direct HIV or immunosuppression effect. The 2-fold higher risk of lung cancer in Indian PHA is likely explained by an increased frequency of smoking [7]. Similarly, marginally significant increases in liver cancer risk (3-fold risk; 95% confidence interval: 0.96–9.26) [13] may be because both HIV and hepatitis viruses B and C are transmitted through needle sharing in drug users and blood product use [43]. Conjunctiva tumor risk could be increased because, for reasons of occupation, ultraviolet light exposure is more common in PHA [9]. The excess risks of other rare tumors are small, and it is difficult to determine if exposures in PHA might be different from those in the general population because their etiologies are not known. Although based on few cases, the 2-fold increase in testicular cancer in Indian PHA [13] is interesting since the incidence of this cancer was, until the mid 1990s, also marginally increased in the developed countries [44]. No risk factors are known to be associated with testicular cancer.

Studies in places like India, where environmental exposures differ greatly in different populations, offer new opportunities to identify novel associations or clarify risk factors. Finding new associations is of general scientific interest. The recent finding of a polyomavirus associated with Merkel cell carcinoma [45], an HIV/AIDS related tumor [11], demonstrates the potential for discovering new viral associations. Careful clinical study of PHA among isolated populations, such as Hill Tribes, might lead novel insights about cancer associations. Similarly, some areas of India have specific parasitic problems (e.g., schistosomiasis or leishmaniasis) that might result in unique clinical manifestations in PHA. The chronic irritation resulting from poor control of such infestations in PHA hypothetically could result in cancer, such as of the bladder or lymphoid organs. There are therefore many opportunities for the alert clinician to generate new observations.

Clearly, prevention of HIV infection must be the highest public health priority in India. For PHA, HIV infection has already occurred. In them, reducing mortality from preventable infections like tuberculosis and Candida is the primary priority. However, success in these goals will reveal new clinical challenges, including from cancer. Thus, India stands at a crossroads where knowledge about cancer in PHA is likely to contribute to understanding cancer itself and may also provide data to develop public health responses to the emergent problem. The renewed focus on HIV/AIDS care in India makes cancer monitoring and research in PHA timely. In this context, systematic gathering of treatment outcome data for cancers in PHA and comparing response and survival to cancers in non-HIV infected population is essential. Such data will allow the development of treatment regimens that might best be suited to PHA in India and permit the investments in antiretroviral therapy to be meaningful to all PHA. Research can provide practical as well as academic benefits, informing both health care and the public health planning. These studies also encourage the development of epidemiology and laboratory capability. Indian researchers should focus on the advantages conferred by undertaking studies that might be uniquely done in their own context and are likely to have public health relevance to India.

## Competing interests

The authors declare that they have no competing interests.

## Authors' contributions

The primary literature was reviewed by RJB. All authors participated in the writing and editing the manuscript.
